# The Impact of Weight Loss during Chemoradiotherapy for Unresectable Esophageal Cancer: Real-World Results

**DOI:** 10.3390/life12050706

**Published:** 2022-05-08

**Authors:** Tzu-Ting Huang, Shang-Yu Chou, Yun-Hsuan Lin, Shau-Hsuan Li, Yen-Hao Chen, Hung-I Lu, Chien-Ming Lo, Fu-Min Fang, Yi-Chun Chiu, Yeh-Pin Chou, Yu-Ming Wang

**Affiliations:** 1 Department of Radiation Oncology & Proton and Radiation Therapy Center, Kaohsiung Chang Gung Memorial Hospital and Chang Gung University College of Medicine, Kaohsiung 83301, Taiwan; tthuang@cgmh.org.tw (T.-T.H.); a9682@cgmh.org.tw (S.-Y.C.); er1122@cgmh.org.tw (Y.-H.L.); fang2569@cgmh.org.tw (F.-M.F.); 2 Department of Computer Science, National Yang Ming Chiao Tung University, 1001 University Road, Hsinchu 30010, Taiwan; 3 Department of Hematology-Oncology, Kaohsiung Chang Gung Memorial Hospital and Chang Gung University College of Medicine, Kaohsiung 83301, Taiwan; lee0624@cgmh.org.tw (S.-H.L.); alex2999@cgmh.org.tw (Y.-H.C.); 4 Department of Thoracic & Cardiovascular Surgery, Kaohsiung Chang Gung Memorial Hospital and Chang Gung University College of Medicine, Kaohsiung 83301, Taiwan; luhungi@cgmh.org.tw (H.-I.L.); t123207424@cgmh.org.tw (C.-M.L.); 5 Division of Hepato-Gastroenterology, Department of Internal Medicine, Kaohsiung Chang Gung Memorial Hospital and Chang Gung University College of Medicine, Kaohsiung 83301, Taiwan; chiuku@cgmh.org.tw (Y.-C.C.); md3@cgmh.org.tw (Y.-P.C.); 6 School of Traditional Chinese Medicine, Chang Gung University, Taoyuan 33302, Taiwan

**Keywords:** esophageal cancer, nutrition, weight loss, concurrent chemoradiotherapy, prognosis

## Abstract

Weight loss is a common phenomenon presented in unresectable esophageal cancer (EC) patients during their definitive chemoradiotherapy (dCRT) treatment course. This study explored the prognostic value of weight changes during dCRT in unresectable EC patients. From 2009 to 2017, 69 cT4b thoracic EC patients undergoing complete curative dCRT without baseline malnutrition were included. Clinical factors were analyzed via the Cox proportional hazards model and survival was analyzed by the Kaplan–Meier method. During dCRT, the median weight loss percentage was 5.51% (IQR = 2.77–8.85%), and the lowest body weight was reached at 35 days (IQR = 23–43 days). Median OS of these patients was 13.5 months. Both univariate and multivariate analysis demonstrated that weight loss ≤ 4% during dCRT was significantly associated with superior OS with a hazard ratio of 2.61 (95% CI: 1.40–4.85, *p* = 0.002). The median OS for patients with weight loss ≤ 4% and >4% during dCRT was 59.6 months and 9.7 months, respectively (*p* = 0.001). Our study demonstrated that weight loss ≤ 4% during dCRT course is a favorable prognostic factor for cT4b EC patients. This index could serve as a nutrition support reference for unresectable EC patients receiving dCRT in the future.

## 1. Introduction

Nutritional status is critical for esophageal cancer (EC) patients. Approximately 60–80% of EC patients are malnourished at diagnosis due to anorexia, dysphagia, reduced food intake, neuroendocrine changes, and elevated systemic inflammation induced by the tumor [1,2,3]. Baseline malnutrition in EC patients may lead to increased treatment-related toxicity [4], treatment interruption [5], more postoperative morbidity, and even worse overall survival [6,7,8,9].

In addition to baseline malnutrition, body weight loss (BWL) during cancer treatment is another important assessment tool for EC patients [10]. Although various tools have been proposed for nutritional status assessment [10,11,12], body weight loss has advantages as it is a simple, objective, and common method, which can serve as a surrogate for nutritional status during treatment.

Definitive chemoradiotherapy (dCRT) is a standard treatment modality for locally advanced and unresectable EC [13]. During the dCRT course, patients’ body weight may further deteriorate due to treatment-related toxicities such as esophagitis and anorexia [14,15,16]. Several studies have reported that BWL during chemoradiotherapy is correlated with poor survival in lung [17], head and neck [18], and rectal cancer patients [19]. However, the relationship between BWL during dCRT and treatment outcomes remains unclear for locally advanced EC patients. An optimal goal of nutrition support for these patients during their dCRT course has yet to be established. Therefore, we conducted this study to investigate the impact of BWL during dCRT on survival for cT4b EC patients without baseline malnutrition and to explore the optimal goal of body weight maintenance.

## 2. Materials and Methods

### 2.1. Patients

From 1 January 2009 to 31 December 2017, a total of 1120 consecutive patients with histologically proven thoracic EC were diagnosed and treated at our institution. Among this group, patients with thoracic cT4b EC who underwent curative dCRT were first selected for this retrospective review. Then, patients with a history of malignant disease, synchronous malignancy, or distant metastasis at an initial staging workup were excluded. Since this study was designed to evaluate nutritional changes during dCRT, patients with pre-treatment body mass index < 18.5 kg/m^2^ [10], body weight nadir in the first week of dCRT, and incomplete radiotherapy treatment course were also excluded for the following analyses, to avoid possible confounding on outcomes [20,21]. Our Institutional Review Board has approved this retrospective study.

### 2.2. Pretreatment Workup

A series of examinations including esophagogastroduodenoscopy (EGD) with biopsy, endoscopic ultrasound (EUS), and chest-computed tomography (CT) scans were performed for all patients at the time of initial diagnosis workup. Bronchoscopy was applied if clinical tracheobronchial tree invasion was suspected. For most patients diagnosed after late 2011, 18F-fluorodeoxyglucose (FDG) positron emission tomography/CT (PET/CT) was included in the initial staging workup. The diagnostic criteria for cT4b EC included tumor invading the great vessels, tracheobronchial tree, or vertebral bodies. Great vessel invasion was defined as the angle of fat plane obliteration by the tumor exceeding 90 degrees on contrasted chest CT, and EUS showing direct tumor invasion into the vessels. Tracheobronchial tree invasion was diagnosed when an irregular/deformed trachea or main bronchus caused by the tumor was identified on contrasted chest CT or direct invasion of the tracheobronchial tree was demonstrated by bronchoscopy. A multidisciplinary esophageal cancer team consisting of medical oncologists, surgical oncologists, and radiation oncologists was formed to discuss patient staging, treatment strategies, and post-treatment evaluation. The 7th edition of the American Joint Committee on Cancer (AJCC) Staging System [22] was used to determine the clinical stage.

### 2.3. Body Weight Measurement and Nutritional Counseling

The baseline body weight and height were measured before the first treatment using digital scales. During the dCRT course, body weight was recorded every week. Maximum weight change was calculated by baseline body weight minus lowest body weight during dCRT, divided by baseline body weight. Two sessions of nutritional counseling were scheduled for all patients; one was arranged after diagnosis and the other was arranged in the late period of dCRT by a licensed nutritionist.

### 2.4. Treatment

Our treatment and follow-up protocols for esophageal cancer have been reported [23], and are briefly summarized here. All patients underwent concurrent chemoradiotherapy with cisplatin (75 mg/m^2^; 4 h infusion; on day 1) and 5-fluorouracil (1000 mg/m^2^; continuous infusion on days 1–4) every 4 weeks. For patients with impaired renal function, defined as creatinine clearance rate <60 mL/min, Carboplatin was used instead.

For radiotherapy, the prescribed dose was 50–50.4 Gy in 25–28 daily fractions, 5 days per week. Gross tumor volumes (GTVs) were delineated by the gross tumor and lymph nodes based on chest CT scan and PET-CT studies. The clinical target volume (CTV) included the GTV with comprehensive regional lymph drain coverage; then, a three-dimensional expansion with 0.5–1.0 cm was used to generate planning target volume (PTV). Additional 10–16 Gy in 5–8 daily fractions were prescribed for patients with gross supraclavicular fossa lymph nodes metastasis. Most treatment plans used intensity-modulated radiation therapy (IMRT) with a 6- or 10 MV photon beam.

### 2.5. Follow-Up Schedule

Weekly outpatient clinic visit was arranged for all patients during dCRT course to monitor body weight change, treatment toxicities, and their general condition. The first follow-up after dCRT completion was arranged 6 weeks later and every 3–4 months for the first two years, then every 6 months for the next 3 years, then once each year. Follow-up workup included toxicity evaluations, physical examinations, chest CT images, EGD with biopsies, and/or PET/CT studies. Treatment toxicity was evaluated by the Common Toxicity Criteria for Adverse Events (CTCAE) ver. 4.0 [24]. Response Evaluation Criteria for Solid Tumors (RECIST) ver. 1.1 [25] was used to evaluate the clinical tumor response based on the first two follow-up chest CT scans within 4 months after completion of dCRT treatment. Clinical complete response (CR) was defined as no detectable residual tumor/ulceration with negative biopsy upon EGD examination, no regional lymph nodes with a short axis ≥ 10 mm in diameter, and no distant metastasis on chest CT studies. Metabolic complete response was defined as a physiologic level of FDG uptake on the primary tumor and regional lymph nodes.

### 2.6. Statistical Analysis

Overall survival (OS) was estimated by the Kaplan–Meier method with the log-rank test for significance. Univariable and multivariable analyses were performed using the Cox proportional hazards model. The optimal cut point for body weight change [26,27], data processing, and computation were performed by R, version 4.0.2 (The R Foundation for Statistical Computing, Vienna, Austria) and SPSS, version 22.0, software (SPSS, Chicago, IL, USA). A two-sided *p*-value lower than 0.05 was considered statistically significant.

## 3. Results

### 3.1. Patient Characteristics

A total of 69 patients were finally included in this study, the median age was 58.5 years, 98.6% patients were male, and 98.6% were diagnosed with squamous cell carcinoma. Over 90% of patients had a history of smoking and alcohol drinking. All patients had regional lymph node metastasis and two-thirds of them (45/69) had cN2-3 disease. The complete patient characteristics are listed in Table 1.

### 3.2. BMI, Body Weight Change, Optimal Cut Point for Weight Change, and Feeding Condition

The mean pretreatment BMI of the patients was 22.8 ± 3.4 kg/m^2^ (range: 18.5–32.3). Sixty-two patients (89.9%) experienced weight loss throughout dCRT. The median percentage of weight loss during dCRT was 5.5% (interquartile range (IQR) = 2.77–8.85%) and the lowest weight was reached at median 35 days (IQR = 23–43 days) after dCRT. The optimal cut point for weight change was 3.61%. For simplicity, we rounded off this number at 4%. Twenty-nine patients (42.0%) maintained oral intake throughout the treatment course, 34 patients (49.3%) had nasogastric (NG) tube intubation, and 6 patients (8.7%) had jejunostomy tube placement before dCRT started.

**Table 1:** Demographics (N = 69).

Characteristic	Value
Age	
Mean	58.5 ± 10.0 (38.8–78.7)
≤65	50 (72.5%)
>65	19 (27.5%)
Gender	
Male	68 (98.6%)
Female	1 (1.4%)
BMI	
Mean	22.8 ± 3.4 (18.5–32.3)
Comorbidities	
Hypertension	17 (24.6%)
Diabetes mellitus	5 (7.2%)
COPD	4 (5.8%)
Liver disease	
Cirrhosis	6 (8.7%)
Chronic HBV	5 (7.2%)
Chronic HCV	2 (2.9%)
Chronic renal disease	1 (1.4%)
Personal history	
Smoking	63 (91.3%)
Betel nut use	42 (60.9%)
Alcohol use	63 (91.3%)
Feeding route	
Oral	29 (42.0%)
Nasogastric tube	34 (49.3%)
Jejunostomy tube	6 (8.7%)
Histology type	
SCC	68 (98.6%)
Adenosquamous	1 (1.4%)
Adenocarcinoma	0 (0.0%)
Clinical lymph node category	
1	24 (34.8%)
2	30 (43.5%)
3	15 (21.7%)
Tumor location	
Upper thoracic esophagus	21 (30.4%)
Middle thoracic esophagus	31 (44.9%)
Lower thoracic esophagus	17 (24.7%)
Tumor invasion site	
Great vessels	28 (40.6%)
Airway	23 (33.3%)
Both	18 (26.1%)
Tumor length	
≤6 cm	33 (47.8%)
>6 cm	36 (52.2%)

Values are number (%) or mean ± SD (range). Abbreviations: BMI = body mass index; dCRT = definitive chemoradiotherapy; HBV = Hepatitis B; HCV = Hepatitis C; COPD = chronic obstructive pulmonary disease SCC = squamous cell carcinoma.

### 3.3. Treatment Response, Toxicities, and Survival Outcomes

Treatment responses were observed in 81.2% patients, and the CR rate was 30.4% (21 patients). Four (5.8%) patients developed grade 3 esophagitis, no patient had greater or equal to grade 3 radiation pneumonitis, 5 patients developed tracheo-esophageal fistula, and 1 patient had tracheo-aortic fistula (Table 2).

The median follow-up time was 61.7 months (range: from 2.5 months to 96.3 months). The median OS for the entire cohort was 13.5 months. The 1-year, 3-year, and 5-year OS rates for these patients were 56.4%, 29.5%, and 24.9%, respectively.

**Figure 1:**
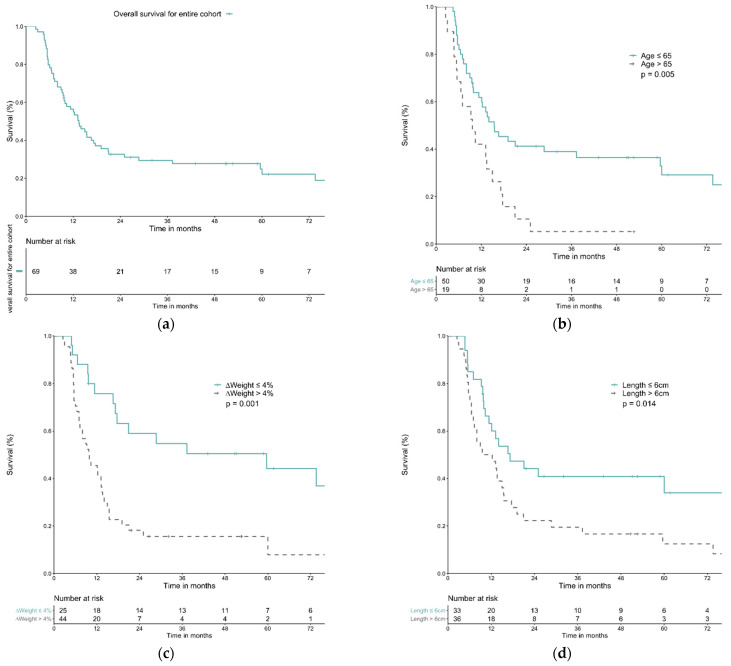
Overall survival curves of patients with T4b esophageal cancer without baseline body mass index < 18.5 kg/m^2^ and OS curves according to different parameters. (**a**) Overall survival curve of the entire cohort. (**b**) Overall survival curve according to age ≤ 65 or >65. (**c**) Overall survival curve according to weight loss during treatment ≤ 4% or >4%. (**d**) Overall survival curve according to tumor length ≤ 6 cm or >6 cm.

Forty-four patients (63.8%) experienced weight loss > 4% during dCRT course. The median OS was 59.6 months for patients with weight loss ≤ 4% and 9.6 months for patients with weight loss > 4%. The OS rate for weight loss ≤ 4% group at 1, 3, and 5 years was 75.8%, 54.7%, 44.2%, respectively, compared with 45.5%, 15.6%, 15.6% in weight loss > 4% group (*p* = 0.001). Only 5 out of 44 patients achieved CR in the weight loss > 4% group, while 16 out of 25 patients achieved CR in the weight loss ≤ 4% group (Fisher’s exact test, *p* = 0.000). In both univariable and multivariable analysis, age ≤ 65 years old (hazard ratio (HR) 1.91, 95% confidence interval (CI) 1.06–3.44, *p* = 0.032), weight change ≤ 4% (HR 2.61, 95% CI 1.40–4.85, *p* = 0.002), and tumor length ≤ 6 cm (HR 1.83, 95% CI 1.05–3.22, *p* = 0.035) were significant favorable prognostic factors (Figure 1 and Table 3).

## 4. Discussion

Our study demonstrated that maintaining a BWL less than 4% during dCRT course is critical for survival in cT4b EC patients. The 5-year OS rate was 44.2% for patients with BWL ≤ 4%, compared to 15.6% for those with BWL > 4%. Both univariable and multivariable analyses confirmed that BWL ≤ 4% was a significant favorable prognostic factor. Furthermore, BWL ≤ 4% during dCRT course was also significantly associated with clinical complete response status, a known positive prognostic factor for T4b EC patients [23]. Therefore, BWL during dCRT is a crucial yet adjustable factor in cT4b EC treatment.

Here, we must emphasize that BWL during dCRT, a dynamic change in nutritional status, differs from baseline nutrition status such as pretreatment BMI, a widely reported prognostic factor for EC patients [8,9,12,20,21,28]. Therefore, in this study, we excluded patients with pretreatment BMI < 18.5 kg/m^2^ in our cohort to avoid the potential confounding effect and aimed at an adjustable factor: changes in body weight during dCRT for EC patients. To our knowledge, this is the first report in the literature to demonstrate the association between body weight change during dCRT and survival for EC patients.

Since BWL was the only modifiable prognosticator for cT4b EC patients found in our institutional analysis, and previous studies have demonstrated that intensive nutritional management could improve nutritional status for EC patients during their dCRT course [29,30,31], a nutritional support program for EC patients has been embedded into our institution multi-modality treatment protocol for EC patients to prevent BWL since the late 2010s. This nutritional support program includes two primary strategies. The first strategy is to establish a pre-treatment enteral feeding route for reliable nutritional intake, preferably feeding jejunostomy, and a nasogastric tube. In a recent prospective multi-center randomized clinical trial, enteral nutrition was proven to alleviate BWL, reduce serum albumin/hemoglobin decline, and decrease grade 3/4 leukopenia rate during dCRT for unresectable EC patients. A nutrition formula with high-proteins and polyunsaturated fat was suggested, while enteral immunonutrition might further maintain nutritional status for EC patients during dCRT course [32,33]. The second strategy was to arrange at least two planned sessions of nutritional consultation by certified clinical nutritionists during the treatment course. The first session was arranged soon after diagnosis for an evaluation of patients’ baseline nutritional status and to design a personalized nutritional support plan based on individual nutritional status, feeding condition, and physical activities. The second session was arranged around the 4th week during dCRT to adjust patients’ personalized nutritional support plans. Early and periodic nutrition assessments/interventions had been shown to improve survival outcomes and significantly reduce weight loss, de-crease unplanned hospital admissions, and increase RT completion rates for EC patients who underwent dCRT [12,31].

Although BWL was a critical prognostic factor in our study, the direct mechanism that causes worse survival for patients who have BWL during treatment remains unclear. Several hypotheses had been proposed to explain this association. First, impaired immunity may play a major role. The immune system is critical to tumor development [34] and deficiency of nutrients such as amino acids, vitamins, minerals, and trace elements in patients who suffer from excessive BWL might have a detrimental impact on immune function [35,36,37,38]. Another possible reason is skeletal muscle loss. Loss of skeletal muscle is commonly observed in EC patients after chemoradiotherapy and has been reported as a poor prognostic factor [39,40,41,42]. Moreover, BWL might cause anatomical changes, increase setup error, and alter the dose distribution of radiotherapy [43,44]. All these problems could increase treatment toxicities and reduce accuracy. Finally, the dysregulated inflammatory response in cachectic patients may induce radiation resistance [45,46]. Elevated circulating cytokines such as TNF-α and IL-6 may activate downstream effectors NF-κB and STAT3 and lead to increased radioresistance by producing growth factors and angiogenic factors [47,48,49].

This present study has several limitations that should be addressed. First, as it was a retrospective study, selection bias and missing data exist. Several nutritional assessment biochemical markers/tools, such as serum albumin, fat-free body mass, or patient-generated subjective global assessment, could not be completely collected and were not included in this study. Second, a strict guideline for feeding route, nasogastric tube placement, or feeding jejunostomy did not exist during the study period. The choice of feeding route largely depended on patients’ preference and physicians’ choice. Third, patients with baseline malnutrition were not included in this study; the optimal body-weight monitoring index for this group of patients remains unclear. Lastly, whether our nutritional support protocol may improve clinical outcomes remains unclear due to the short follow-up time and limited case numbers. A larger cohort and long-term follow-up are warranted to confirm the potential benefits of our protocol.

## 5. Conclusions

Maintaining a cT4b EC patients’ BWL of less than 4% of their initial body weight during dCRT is a significant prognostic factor for OS and could serve as an individualized goal for nutritional support. Future prospective studies are warranted to validate our findings.

## Figures and Tables

**Table 2:** Summary of dCRT treatment.

Variable	No. (%)
Weight change during	
≤4%	25 (36.2%)
>4%	44 (63.8%)
RT modality	
3D-CRT	1 (1.4%)
2D + IMRT	12 (17.4%)
IMRT	56 (81.2%)
Post-dCRT complication	
≥Grade 3 esophagitis	4 (5.8%)
≥Grade 3 radiation pneumonitis	0 (0%)
Tracheo-esophageal fistula	5 (7.2%)
Tracheo-aortic fistula	1 (1.4%)
Treatment response	
Complete response	21 (30.4%)
Partial response	35 (50.8%)
Stable/progression disease	9 (13.0%)
Non-accessible	4 (5.8%)

Abbreviations: dCRT = definitive chemoradiotherapy; RT = radiation therapy; 3D-CRT = 3-dimensional conformal RT; IMRT = intensity-modulated RT.

**Table 3:** Overall/median survival and univariable/multivariable analysis of clinical parameters predicting overall survival.

Variable	N	OS (%)			MS (Months)	*p* Value	Univariable Analysis			Multivariable Analysis		
		1-Year	3-Year	5-Year			HR	95% CI	*p* Value	HR	95% CI	*p* Value
All	69	56.4%	29.5%	24.9%	13.5							
Age												
≤65	50	61.8%	38.9%	32.8%	15.4	0.005 *	2.28	1.26–4.10	0.006 *	1.91	1.06–3.44	0.032 *
>65	19	42.1%	5.3%	5.3%	9.6							
Weight change												
≤4%	25	75.8%	54.7%	44.2%	59.6	0.001 *	2.83	1.53–5.26	0.001 *	2.61	1.40–4.85	0.002 *
>4%	44	45.5%	15.6%	15.6%	9.7							
Clinical N stage												
cN1	24	70.6%	52.9%	45.4%	59.6	0.010 *	2.22	1.19–4.13	0.012 *	N.S.		
cN2-3	45	48.9%	16.9%	14.1%	11.4							
Tumor location												
U/3 EC	21	57.1%	23.8%	23.8%	13.2	0.377	N.S.			N.S.		
M/3 EC	31	58.1%	41.4%	31.0%	16.6							
L/3 EC	17	51.8%	12.9%	12.9%	12.1							
Tumor invasion												
Great vessels	28	60.3%	22.6%	18.1%	13.5	0.457	N.S.			N.S.		
Airway	23	65.2%	38.6%	30.9%	17.6							
Both	18	38.9%	27.8%	27.8%	8.0							
Tumor length												
≤6 cm	33	63.1%	40.8%	40.8%	17.2	0.014 *	1.98	1.13–3.46	0.016 *	1.83	1.05–3.22	0.035 *
>6 cm	36	50.0%	19.4%	12.5%	9.5							

Abbreviations: N = number; OS = overall survival rate; MS = median survival; HR = hazard ratio; CI = confidence interval; BMI = body mass index; U/3 = upper third; M/3 = middle third; L/3 = lower third; EC = esophageal cancer; * = statistically significant; N.S = not statistically significant.

## Data Availability

The data presented in this study are available on request from the corresponding author. The data are not publicly available due to the nature of this research; participants of this study did not agree for their data to be shared publicly.
